# Compliant gluten-free children with celiac disease: an evaluation of psychological distress

**DOI:** 10.1186/1471-2431-11-46

**Published:** 2011-05-27

**Authors:** Luigi Mazzone, Laura Reale, Massimo Spina, Manuela Guarnera, Elena Lionetti, Serena Martorana, Domenico Mazzone

**Affiliations:** 1 Department of Paediatrics. Division of Child Neurology and Psychiatry. University of Catania, Catania, Italy; 2 Department of Paediatrics, University of Catania, Catania, Italy

## Abstract

**Background:**

Children with chronic illnesses are known to have increased risks for emotional and behavioral problems. In the present study, children and adolescent suffering from celiac disease (CD) were compared with healthy controls to assess differences in the psychological profile.

**Methods:**

A total of 100 well-treated and compliant CD patients (65 females/35 males; age mean ± SD: 10.38 ± 2.71) were compared to 100 normal controls (58 females/42 males; age mean ± SD: 11.47 ± 2.61). Emotional and behavioral problems were assessed by the Child Behavior Checklist (CBCL), the Children's Depression Inventory (CDI) and the Multidimensional Anxiety Scale for Children (MASC).

**Results:**

Subjects with CD self-reported an increased rate of anxiety and depression symptoms and showed higher scores in "harm avoidance" and "somatic complaints", in the CBCL parent-report questionnaire, as compared to healthy control subjects. Furthermore, gender differences could be observed in the group of CD patients, with males displaying significantly higher CBCL externalizing scores, in social, thought and attention problems, as compared to female, who in turns showed more prominent internalizing symptoms such as depression.

**Conclusions:**

The increased rate of emotional and behavioral problems in children and adolescent with CD emphasizes the importance of an early detection of mental health problems in these children.

## Background

Celiac disease (CD) is an autoimmune enteropathy characterized by intolerance to dietary gluten. The clinical spectrum of celiac disease is extremely wide, varying in onset, duration and severity of the disease, and the compliance to a gluten-free diet is also extremely variable. Besides the classic form, which shows the typical gastrointestinal manifestations, there are also atypical and asymptomatic (silent) forms of celiac disease [1-3].

Celiac disease can also be found in association with other autoimmune diseases (i.e. diabetes, thyroiditis of Hashimoto, psoriasis), as well as with extra-intestinal complications such as neurologic and psychiatric disturbances, which may either follow or precede symptoms and diagnosis of the celiac disease [4-6]. Several studies have documented the occurrence of internalizing disorders such as depression, anxiety, and psychoneurotic symptoms in adults with celiac disease, before or after the introduction of a gluten-free diet and food restriction [7-11]. Children with celiac disease can also suffer from neurological and psychological disorders, including headaches, attention-deficit/hyperactivity disorder (ADHD), learning and tic disorders, depression and anxiety, mostly before any dietary treatment [12-15]. An association between autism and CD has also been reported, although a direct link still has to be determined [16,17]. Recently, another study suggested the existence of a low prevalence of neurological and psychiatric disorders such as febrile seizures, epilepsy, headache, mental retardation, neuropathy, and bipolar disorder in children with gluten sensitivity [18].

The pathogenesis processes responsible for the neurological complications in celiac disease still remain poorly understood [19], even if several mechanisms have been proposed. Indeed, some studies have hypothesized a role for the deficit of folic acid, vitamin E, and biopterin, documented in these patients, whereas other studies have identified crossreacting antibodies, immune complex disease, and direct toxicity as putative key players [20,21]. Recently, additional factors have been suggested, including brain perfusion abnormalities [22], which have been observed in the superior and anterior areas of the frontal cortex and anterior cingulated cortex in patients with celiac disease [20]. An alteration of perfusion in the same brain areas has also been reported in patients with neurological and psychiatric disorders, including depression and anxiety, thus providing a possible explanation for the association between the two conditions [23,24].

Since gluten-free or vitamin-supplemented diets were introduced for the treatment of these patients, several studies have tried to investigate the effects of these specific dietary regimens on the neurological and psychological aspects associated with celiac disease, providing somewhat conflicting results [25-27]. For instance, in one study, the beginning of a standard gluten-free diet, or a vitamin B-6-supplemented gluten-free diet was shown to improve internalizing symptoms in adults [25]. In line with this, another study reported that ADHD-like symptoms were markedly overrepresented among untreated CD patients (age range: 3 to 57) and that a gluten-free diet improved symptoms within a short period of time [26]. Finally, the institution of a gluten-free diet in a group of children with CD was also shown to improve neurological symptoms such as headaches in 77% of the cases [12]. Conversely, it has also been described that depressive symptoms can arise after the introduction of a gluten-free diet [27]. However, so far, only a few reports have described the pattern of behavioral and emotional characteristics in CD children after the introduction of a gluten-free diet.

In the present study, we evaluated the emotional and behavioral profiles in a sample of children suffering from celiac disease undergoing a strict gluten-free regimen, to try to identify peculiar psychological features, both as compared to healthy peers and within the CD group itself according to gender.

## Methods

### Patients and controls

A total of 100 children (65 females/35 males, aged 7 to 18 years) suffering from celiac disease were included in the study. Diagnosis of celiac disease was aided by the following serologic assays: IgA and IgG anti-gliadin antibody (AGA), IgA-class EMA and IgA-class anti-tTG antibodies in the serum of these patients. Antiendomysium antibodies (EMA) are evaluated by indirect immunofluorescence assay in umbilical cord substrate, as previously described [28,29]. All subjects were hospitalized at the time of diagnosis, submitted to a strict gluten-free diet and checked every six months. Self-reported adherence to the gluten-free diet was good in 95% of patients, with only 5% reporting being on a fairly strict gluten-free regimen. The histological analysis of biopsy specimens obtained from the distal part of the duodenum revealed the presence of a gluten sensitive enteropathy, according to the ESPGHAN criteria [1].

A total of 100 age-matched control children (58 females/42 males) were randomly selected from a database of healthy children attending a well-being pediatric clinic for normal developmental check-up. Control subjects were also tested for the presence of anti-gliadin antibody, anti-endomysial antibody, and anti-tissue transglutamine antibody. The socioeconomic status of the families was determined using different indicators, including parental educational level, parental occupational level and family's availability of material resources.

Inclusion criteria for CD patients were the following:

- clinical and laboratory diagnosis of CD

- absence of mental retardation

- lack of relevant psychopathological disorders in the family.

The present study was approved by the institutional review board of ethics committee at the "Policlinico Institute" of University of Catania, Catania, Italy. At the time of enrollment in the study, mothers and adolescents gave written consent and assent, respectively.

### Assessment of behavioural and emotional problems

The Multidimensional Anxiety Scale for Children (MASC), 39-item four point Likert-style self-report scale, was used to measure anxiety symptoms: physical symptoms, harm avoidance, social anxiety and separation anxiety were evaluated. Raw scores were converted into standard T-scores, and T-score >65 indicated the presence of anxiety symptoms. The MASC was also completed by the children [30].

The Child Behaviour Checklist (CBCL), a 113-item questionnaire, was completed by the parents of CD patients. The CBCL is used to rate children behavior and emotional problems, both globally and along the two dimensions of internalizing symptoms (anxiety and depression), and externalizing symptoms (aggression and hyperactivity). Raw scores for each clinical factor were transformed into T-scores based on published norms. T-scores ≥ 70 were considered indicative of clinical impairment [31,32].

The Children's Depression Inventory (CDI), completed by the child, was used to rate depression symptoms. This scale consists of 27 items scored on a three-point scale (0 absent, 1 moderate and 2 severe) indicating the growing severity of symptoms. A 19-point cut-off indicates the ideal threshold for discriminating children at risk of depression from healthy children [33].

The presence of autistic disorders in our patients was assessed by evaluating the criteria outlined in the DSM-IV-TR [34]. In the CD group, the neuropsychological evaluation was conducted 6-9 months after the diagnostic biopsy.

### Data analysis

Statistical analyses were performed using the Statistical Package for Social Sciences (SPSS 14.0 for Windows). Both descriptive and inferential analyses were undertaken, including Chi square test, Student's t-test and non-parametric tests. To investigate gender differences within the CD group the Chi-square test and the Fisher's exact test were also performed. An alpha level of .05 was used for all statistical tests.

## Results

### Clinical characteristics

The present study included 200 children and adolescents. Among them, 100 (35 males/65 females, age mean ± SD: 10.38 ± 2.71) suffered from celiac disease and 100 (42 males/58 females, age mean ± SD: 11.47 ± 2.61) were included in the normal control (NC) group. The demographic and clinical characteristics of the sample are summarized in Table 1. The CD and NC groups were similar for age and gender distribution, as well as for the socioeconomic status of their families. Among children with celiac disease, 25 suffered from somatic complaints including headache (19 children out of 100), stomachache (9/100) or musculoskeletal pains (2/100). Furthermore, two children in the CD group could be classified within the spectrum of autistic disorders, according to DSM-IV-TR.

**Table 1:** Demographics and clinical characteristics of the sample

Groups	Celiac Subjects (N = 100)	CTRL (N = 100)	***p***^**a**^
Males/females	35/65	42/58	NS
Age (mean ± SD)	10.38 ± 2.71	11.47 ± 2.61	NS
Age at onset (mean ± SD)			
Male	3.51 ± 2.67	-	-
Female	4.66 ± 4.39	-	-

^a ^NS indicates p > 0.05.

### Emotional and behavioral profile

**Figure 1:**
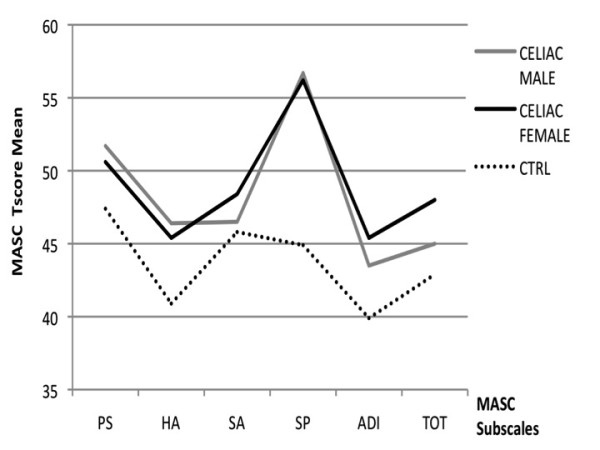
**MASC profile in celiac (males and females) and normal control subjects**. CTRL = normal controls; PS = physical symptoms; HA = harm avoidance; SA = social anxiety; SP = separation anxiety; ADI= Anxiety Disorder Index; TOT = Total

**Figure 2:**
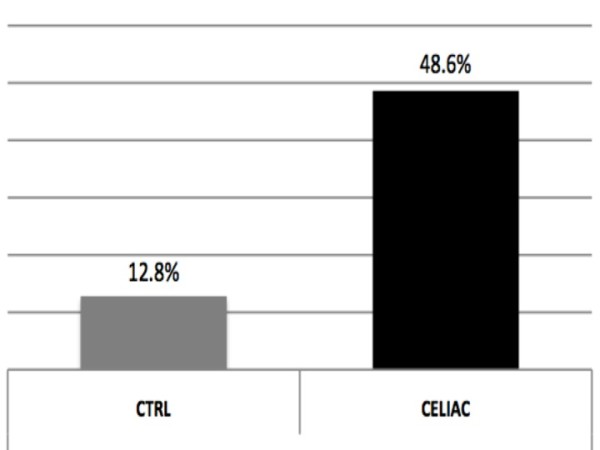
**Percent of subjects with MASC scores in the pathological range in control and celiac groups**. CTRL = normal controls; T-score > 65 was considered as cut-off for pathological range

CD children showed significantly higher scores in the two completed self-rating scales (MASC and CDI) as compared to normal controls: 50.0 ± 8.3 vs. 42.9 ± 6.6 (p < 0.01) for total MASC, and 8.1 ± 5.7 vs. 5.6 ± 3.4 (p < 0.01) for CDI scale, respectively; MASC subscales "harm avoidance" and "separation panic" also revealed higher scores for CD children (44.8 ± 9.3 vs. 41.9 ± 9.2; p = 0.028 and 55.0 ± 10.7 vs. 48.7 ± 8.7; p < 0.01, respectively, for the comparison with NC) (Table 2; Figure 1). Although, according to the established norms, most of the patients had scores within the non-pathological range, a higher proportion of CD patients had pathological MASC total scores (T-score > 65) as compared to NC (48.6% vs. 12.8%; p < 0.05) (Figure 2).

**Table 2:** Average psychological and behavioral scores in celiac and control groups

Groups	Celiac Subjects (N = 100)	CTRL (N = 100)	***p***^**f**^
CBCL Total^a^	51.2 ± 9.7	51.1 ± 9.2	NS
CBCL Int^b^	51.4 ± 9.4	52.3 ± 8.4	NS
CBCL Ext^c^	49.0 ± 8.9	51.8 ± 8.5	NS
CDI^d^	8.1 ± 5.7	5.6 ± 3.4	<0.01
MASC^e^	50.0 ± 8.3	42.9 ± 6.6	<0.01

^a ^Parent Child Behavior Checklist Total Score (T-scores); ^b ^Parent Child Behavior Checklist Internalizing Score (T-scores); ^c ^Parent Child Behavior Checklist Externalizing Score (T-scores); ^d ^Children's Depression Inventory (raw scores); ^e ^Multidimensional Anxiety Scale for Children (T-scores); ^f ^NS indicates P > 0.05.

The CBCL analysis, completed by the parents, failed to show any significant differences in total, internalizing and externalizing scores between the two groups; however, the subscale "somatic complaints" revealed higher scores for CD patients than for NC (57.5 ± 7.6 vs. 54.6 ± 6.6; p = 0.004) (Table 2)

The results of the neuropsychological tests were also analyzed in relation to the elapsed time since diagnosis of CD: an inverse correlation was observed with CDI and MASC total scores (CDI: r = -0.26; P = 0.03; MASC: r = -0.24; P = 0.04), but not with CBCL total and subscale scores (CBCL tot: r = -0.14; P = 0.25; CBCL int: r = -0.23; P = 0.06; CBCL ext: r = -0.07; P = 0.55).

### Gender difference within CD group

**Figure 3:**
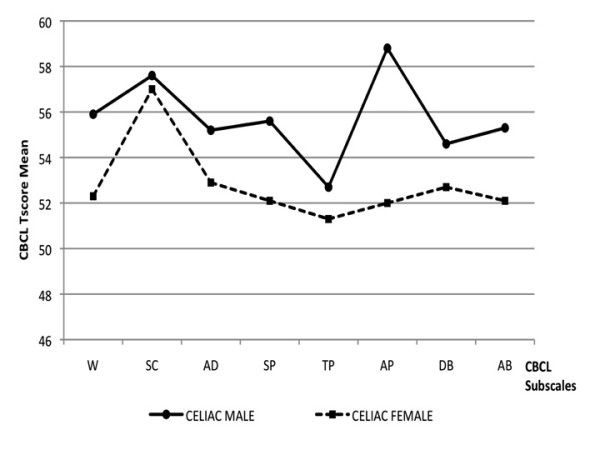
**CBCL profile in celiac group: gender difference**. W = Withdrawn; SC = Somatic Complaint; AD = Anxious/Depressed; SP = Social Problems; TP = Thought Problems; AP = Attention Problems; DB = Delinquent Behavior; AB = Aggressive Behavior

The analysis of the psychological profiles according to the gender revealed significant differences between males and females within the CD group (Table 3). CD males showed significantly higher scores for total CBCL externalizing items (52.3 ± 8.2 vs. 47.9 ± 8.7; P = 0.016), as well as for the CBCL sub-items "withdrawn" (56.3 ± 10.2 vs. 52.9 ± 5.1; p = 0.028), "social" (55.2 ± 6.9 vs. 52.5 ± 4.1; p = 0.016), "thought" (53.7 ± 5.6 vs. 51.8 ± 3.6; p = 0.042), and "attention" problems (57.3 ± 7.0 vs. 52.3 ± 3.9; p < 0.01), as compared to CD females (Table 3; Figure 3). By contrast, CD females showed an increased rate of anxiety and depression symptoms, as indicated by significantly higher MASC Total (48.6 ± 8.6 vs. 45.5 ± 8.9; p = 0.013) and CDI (7.9 ± 5.5 vs. 5.6 ± 3.4; p < 0.01) scores (Figure 1).

**Table 3:** Emotional and behavioral profile: gender differences in the celiac group

Groups	Celiac Females (N = 65)	Celiac Males (N = 35)	***p***^**f**^
			

CBCL Total^a^	49.4 ± 9.2	53.6 ± 11.7	NS
CBCL Int^b^	50.3 ± 8.5	53.9 ± 10.9	NS
CBCL Ext^c^	47.9 ± 8.7	52.3 ± 8.2	0.016
CBCL *subitems:*			
*Withdrawn*	52.9 ± 5.1	56.3 ± 10.2	0.028
*Somatic Complaint*	57.0 ± 7.0	57.6 ± 7.6	NS
*Anxious/Depressed*	52.9 ± 4.8	55.2 ± 6.3	NS
*Social Problems*	52.5 ± 4.1	55.2 ± 6.9	0.016
*Thought Problems*	51.8 ± 3.6	53.7 ± 5.6	0.042
*Attention Problems*	52.3 ± 3.9	57.3 ± 7.0	< 0.01
*Delinquent Behavior*	52.7 ± 4.4	54.6 ± 5.4	NS
*Aggressive Behavior*	52.4 ± 4.5	54.5 ± 6.2	NS
			
MASC Total^d^	48.6 ± 8.6	45.5 ± 8.9	0.013
			
CDI^e^	7.9 ± 5.5	5.6 ± 3.4	<0.01

^a ^Parent Child Behavior Checklist Total Score (T-scores); ^b ^Parent Child Behavior Checklist Internalizing Score (T-scores); ^c ^Parent Child Behavior Checklist Externalizing Score (T-scores); ^d ^Multidimensional Anxiety Scale for Children (T-scores); ^e ^Children's Depression Inventory (raw scores); ^f ^NS indicates P > 0.05.

## Discussion

Understanding the association between the onset of the illness, as well as the changes in dietary regimens, and the psychological sequels in young patients suffering from CD is a crucial aspect to try to improve the quality of their daily life. In the present study, we focused our attention on the emotional and behavioral profile, to try to identify the psychological symptoms that most likely could arise in this pathological condition. Previous reports have outlined the prevalence of emotional and disruptive behavioral disorders in CD children, which were improved after the introduction of the gluten-free diet [12-15]. Similarly, ADHD symptoms were also shown to decrease significantly by six months of gluten-free regimens [14,15]. Other studies documented the presence of anxious-depressive symptoms in the pre-diet stage of the disease with a decrease of anxiety symptoms, but not of symptoms related to depression, after switching diets [8].

The results shown in this study are partially contradictory with the ones reported in the literature. In our cohort, CD children on a strict gluten-free diet showed higher scores of behavioral and emotional symptoms at 7.41+/-4.08 years after diagnosis and initiation of diet, as compared to healthy control individuals. Furthermore, an increase in depressive and anxiety symptoms from the time of CD diagnosis was also observed, even if no further increases were documented advancing with age. Moreover, most of CD patients still showed significantly higher scores for anxiety, harm avoidance, separation panic and somatic complaints, even after the introduction of dietary regimens [8,14,15]. Likewise, symptoms of depression were significantly higher in CD children as compared to normal controls, with 13.84% females (vs. 8.57% males) exceeding the cut-off. Furthermore, as investigated by CBCL, females with CD showed higher scores for internalizing symptoms (i.e. anxiety) as compared to males, whereas CD males showed significantly higher externalizing scores (including withdrawn, social, thought and attention problems). These findings may suggest that females and males with CD perceive the burden of their disease in a different manner and are in line with the results of a previous study reporting a poorer psychological general well being in female than male [11]. The introduction of a gluten free diet results in a radical change in eating habits and lifestyle of CD children, and it can be hard to accept and stressful to follow. This contributes to induce in most patients a high level of anxiety, which may show in a different way according to the gender susceptibility, resulting in depression in females and aggression and irritability in males [4]. Acceptance of the gluten-free diet is also dependent on the age, as the adaptation to the regimen is particularly problematic for adolescents between 12 to 17 years old, a time in life when the interactions with peers and adults become more difficult. In this context, a strict food regimen can be considered of negative influence on the social life. In line with this, previous studies showed that children and adolescents suffering from other chronic conditions such as migraine, asthma and fibromyalgia, were more anxious and depressed as compared to their healthy peers [35-37]. Taken together, all these reports indicate that the impact of a chronic condition during childhood and adolescence may be difficult to manage, thus representing a risk factor for psychiatric disorders in subjects with psychologic vulnerability. In these delicate conditions the family in a first place, and the social environment in a second, become of crucial importance for the acceptance of the illness: parents should encourage their children not to hide their condition, thus contributing to increase their self-esteem [38,39].

Besides the significant results, the present study shows important limitations and it should be viewed in the context of the following considerations. First, considering that the subjects included in the study were entirely derived from a single university clinic, this sample cannot be assumed as indicative of non-academic setting. Indeed, the sample was clinically referred and not intended to be representative of children with CD in the population. Second, symptoms scores were derived from assessment scales filled out by the parents and self-report questionnaires completed by the children themselves, and a formal diagnosis of psychiatric disorders was not performed (although these rating scales have been shown to be valid instruments to identify children with psychiatric symptoms). Third, even though the social and cognitive relational skills of these patients were evaluated by professional child neuropsychiatrists, however, a complete cognitive evaluation with normative scale was not assessed.

## Conclusion

Besides the limitations outlined above, the results presented here, showing a higher incidence of psychological problems in children suffering from celiac disease under strict food regimens, suggest that these patients may need a psychological support, in order to improve the acceptance of the gluten-free diet, thus limiting the lack of compliance to treatment and the related disease complications. Ideally, this type of psychological intervention should also involve parents and school teachers, which may play an essential role in such diseases in order to promote a good social adaptation [40]. Our previous experience has shown that such a cognitive-behavioral family therapy might be an effective tool for improving emotional and behavioral disorders in children with a chronic and painful disease such as beta-thalassaemia major [41]. Therefore, cognitive behavioral family therapy may also be a useful tool to give support to CD children and their families.

## Competing interests

The authors declare that they have no competing interests.

## Authors' contributions

DM and MS designed the study; LM pulled all the information together and wrote the manuscript; LR and MG performed statistical analysis; SM and EL collected the data

Each author read and approved the final version of the manuscript.

## Pre-publication history

The pre-publication history for this paper can be accessed here:

http://www.biomedcentral.com/1471-2431/11/46/prepub
